# Apigenin Inhibits Growth of Breast Cancer Cells: The Role of ERα and HER2/neu

**Published:** 2015

**Authors:** A. M. Scherbakov, O. E. Andreeva

**Affiliations:** Blokhin N. N. Russian Cancer Research Center, Kashirskoye Shosse, 24, Moscow, 115478, Russia

**Keywords:** breast cancer, phytoestrogens, estrogen receptor, HER2/neu

## Abstract

Phytoestrogens are a group of plant-derived compounds with an estrogen-like activity. In mammalians, phytoestrogens bind to the estrogen receptor (ER) and participate in the regulation of cell growth and gene transcription. There are several reports of the cytotoxic effects of phytoestrogens in different cancer cell lines. The aim of this study was to measure the phytoestrogen activity against breast cancer cells with different levels of ER expression and to elucidate the molecular pathways regulated by the leader compound. Methods used in the study include immunoblotting, transfection with a luciferase reporter vector, and a MTT test. We demonstrated the absence of a significant difference between ER+ and ER– breast cancer cell lines in their response to cytotoxic stimuli: treatment with high doses of phytoestrogens (apigenin, genistein, quercetin, naringenin) had the same efficiency in ER-positive and ER-negative cells. Incubation of breast cancer cells with apigenin revealed the highest cytotoxicity of this compound; on the contrary, naringenin treatment resulted in a low cytotoxic activity. It was shown that high doses of apigenin (50 μM) do not display estrogen-like activity and can suppress ER activation by 17β-estradiol. Cultivation of HER2-positive breast cancer SKBR3 cells in the presence of apigenin resulted in a decrease in HER2/neu expression, accompanied by cleavage of an apoptosis substrate PARP. Therefore, the cytotoxic effects of phytoestrogens are not associated with the steroid receptors of breast cancer cells. Apigenin was found to be the most effective phytoestrogen that strongly inhibits the growth of breast cancer cells, including HER2-positive ones.

## INTRODUCTION

Breast cancer is the most common cancer in females, ranking second in the incidence rate after skin neoplasms in the Russian population [1-4]. The search for new prospective compounds that could inhibit the development of breast cancer and the analysis of their impact on tumor cells is one of the priorities in oncology. Given the important role of hormones in the development of reproductive system tumors, compounds structurally similar to estrogens, e.g., phytoestrogens, are of particular interest. Phytoestrogens are plant-derived compounds with steroid-like structures [5]. Because of their “hormonal” properties, phytoestrogens are also referred to as “food hormones.” Phytoestrogens are unique in their paradoxical effect on cells: depending on the conditions, they can either inhibit tumor growth or act as cell protectors [5-7].

Initially, interest in phytoestrogen research arose from the analysis of epidemiological data showing a reduced rate of tumor incidence and cancer mortality in a number of geographical areas with high consumption of fruits and vegetables [8-10]. A study by Knekt *et al*. [8], which was conducted in Finland, included 9,959 individuals who were followed from 1967 to 1991 and whose individual consumption of phytoestrogens with food was analyzed. A total of 997 cases of cancer (ca. 10% of the complete sample) were identified over the entire period of the study, including 151 cases of lung cancer. A statistical analysis showed that the relative risk of cancers (all localizations) in a group with high consumption of phytoestrogens was reduced to 0.8 (the risk level in a group with low consumption of phytoestrogens was taken as 1). The most significant results were obtained upon analysis of the incidence rate of lung cancer; the risk dropped to 0.54 in the group with high consumption of phytoestrogens [8]. Similar tendencies were found upon examination of 1,031 ovarian cancer females and 2,411 healthy donors in Italy over a period between 1992 and 1999 [11]. According to Rossi* et al. *[11], the risk of ovarian cancer dropped to 0.63 in the group with high consumption of flavonols (in particular, quercetin) and to 0.51 in the group with high consumption of foods rich in isoflavones (e.g., genistein). Therefore, the epidemiological data indicate the advisability of increased consumption of foods rich in phytoestrogens to prevent cancer.

However, the epidemiological data do not reveal the molecular mechanisms by which phytoestrogens affect tumor cells and/or protect normal cells from malignant transformation. This is why an extensive search for the main intracellular targets of these compounds is currently underway in *in vitro *models [12-17]. The key targets of phytoestrogens in tumor cells are believed to be receptor tyrosine kinases, including the epidermal growth factor receptor (EGFR) [18-20], fibroblast growth factor receptor 2 (FGFR2) [21], HER2/ neu [22], vascular endothelial growth factor receptor 3 (VEGFR3) [21, 23], the platelet-derived growth factor receptor alpha and beta (PDGFRα and s) [21], etc. In addition to receptors, some members of the phytoestrogen class effectively inhibit the intracellular kinases involved in the regulation of cell proliferation and cell survival, such as p21-activated kinase 3 (PAK3), phosphatidylinositol 3-kinase (PI3K), Akt, PIM1, Aurora-A, Janus kinase 3 (JAK3), etc. [15, 16, 21]. The wide range of the potential targets of phytoestrogens makes these compounds promising for further experimental and clinical studies.

Is the estrogen receptor (ER) required for the antiproliferative effect of phytoestrogens on tumor cells, and is the hormone-like effect of phytoestrogens concentration- dependent? There is no definite answer to these questions [5, 6, 17]. The aim of this study was to investigate the effect of members of the main groups of phytoestrogens on breast cancer cells with various ER statuses and to analyze the molecular pathways responsible for the antiproliferative and cytotoxic effects of a leader compound. Using human breast cancer cell lines, we demonstrated that the antiproliferative effect of high doses of phytoestrogens (apigenin, genistein, quercetin, naringenin) did not depend on the status of steroid hormone receptors. *In vitro *experiments revealed a similar efficacy of these compounds in a ER-positive MCF-7 cell line and ER-negative SKBR3 model. The maximum antiproliferative effect was observed for flavone apigenin that was analyzed in more detail as the leader compound. An increase in apigenin concentrations from 5 to 50 μM in MCF-7 cells was demonstrated to result in a “switch” from estrogen-like (similar to the effect of 17β-estradiol, a natural ligand of ERα) to anti-estrogenic effects (similar to the effect of antiestrogen drugs): a high apigenin dose inhibited activation of estrogen receptors by 17β-estradiol. ERnegative SKBR3 breast cancer cells are known to be characterized by a high level of HER2/neu, one of the key receptors defining the high aggressiveness and survival of tumor cells [24]. Immunoblotting demonstrated that apigenin at a dose of above 25 μM reduces the expression of HER2/neu in SKBR3 cells, with a simultaneous degradation of the apoptosis effect or substrate poly ADP-ribose polymerase (PARP). Apigenin was the most promising among the tested compounds, demonstrating significant inhibition of growth of breast cancer cells with various ERα statuses, including HER2-positive ones.

## MATERIALS AND METHODS

**Fig. 1:**
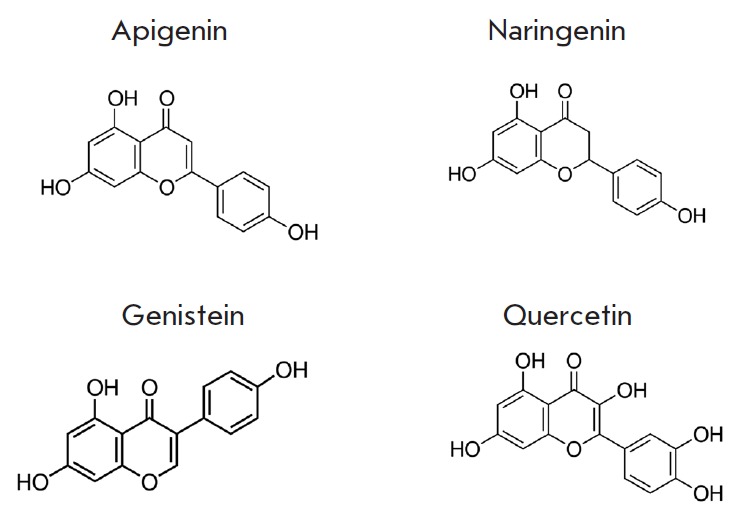
Chemical structures of phytoestrogens (apigenin, naringenin, genistein, quercetin)

Phytoestrogens from various groups were studied: apigenin (flavone), naringenin (flavanone), genistein (isoflavone), and quercetin (flavonol). Quercetin, genistein, and naringenin were purchased from Sigma-Aldrich (USA), apigenin was from Enzo Biochem (USA); the chemical purity of each compound was at least 97%. The chemical structures of the compounds are shown in *Fig. 1*. The compounds were dissolved in dimethylsulfoxide at a concentration of 50 mM, and the solutions were stored until use at –20°C.

Human breast cancer cells MCF-7 (ERα+/HER2–) and SKBR3 (ERα–/HER2+) were obtained from the collection of the Blokhin N.N. Russian Cancer Research Center. The cell lines were cultured *in vitro *in a standard DMEM medium (Biolot, Russia) with 10% fetal calf serum (HyClone, USA) and gentamycin (50 U/mL, PanEco, Russia) at 37 °C, 5% of CO_2_, and a relative humidity of 80–90%. The cell growth rate was determined using a MTT assay, based on the uptake of the MTT reagent (3-[4,5-dimethylthiazol-2]-2,5-diphenyltetrazolium bromide) by the living cells [25, 26].

To determine the transcriptional activity of ERα, the cells were transfected with a plasmid containing the luciferase reporter gene under the control of an ERsensitive promoter (ERE/Luc); the plasmid was a kind gift from George Reid (European Molecular Biology Laboratory, Germany) [27]. The cells were transfected using a Metafectene® PRO reagent according to the manufacturer’s recommendations (Biontex Laboratories, Germany). The efficacy and potential toxicity of the transfection was monitored by co-transfection of the cells with a plasmid containing the β-galactosidase gene. The luciferase activity was calculated in arbitrary units (ratio of the total luciferase activity to the galactosidase activity in samples).

For immunoblotting purposes, the cells at 80% confluency were detached from the dishes (60 mm, Corning, USA) into 1 mL of a phosphate buffer. To obtain a total cell extract, samples were added with 130 μL of the following buffer: 50 mM Tris-HCl pH 7.4, 1% SDS (sodium dodecyl sulfate), 1% Igepal CA-630, 0.25% Na deoxycholate, 150 mM NaCl, 1 mM EDTA (ethylenediaminetetraacetic acid), 1 mM PMSF (phenylmethanesulfonyl fluoride); 1 μg/mL of aprotinin, leupeptin, and pepstatin; and 1 mM Na orthovanadate and 1 mM NaF. Total cell extracts were sonicated on a SoniPrep 150 Plus disintegrator (MSE) (five cycles of 10 s each with an amplitude of 3.2) to reduce the viscosity of a solution. Cell extract samples were then centrifuged (10,000 *g*, 10 min, +4oC, Eppendorf 5417R centrifuge, Germany), and standard electrophoresis and immunoblotting procedures were performed. The levels of HER2/neu and PARP were determined by primary antibodies (Cell Signaling Technology, USA). Antibodies to β-actin (Cell Signaling Technology, USA) were used to monitor the effectiveness of immunoblotting and to normalize the results. Detection was performed using secondary horseradish peroxidase-conjugated antibodies (Jackson ImmunoResearch, USA) in the LAS 4000 system (GE HealthCare, USA). DATAPLOT software (USA) was used for statistical analysis. In all cases, the statistical criteria were considered to be significant at *p* < 0.05; each experiment was performed at least in triplicate.

## RESULTS AND DISCUSSION

**Comparison of the cytotoxic properties of various groups of phytoestrogens with respect to breast cancer cells: selection of the leader compound**

**Fig. 2:**
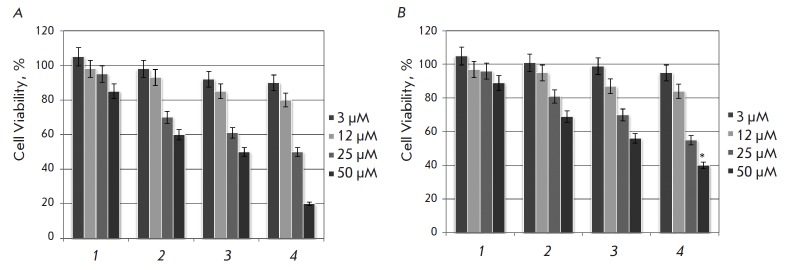
Cytostatic effect of phytoestrogens on breast cancer cells MCF-7 (A) and SKBR3 (B). Data of a MTT test conducted after 3-day cell growth in the presence of phytoestrogens: 1 – naringenin, 2 – genistein, 3 – quercetin, 4 – apigenin. The chart shows the number of living cells after treatment with phytoestrogens. The number of control cells of an appropriate cell line is taken as 100%. ^*^p < 0.05 compared to the number of MCF-7 cells survived at an apigenin dose of 50 μM

At the first stage of the study, the antiproliferative effect of high doses of phytoestrogens was evaluated in the MTT test. ERα-positive cells of the MCF-7 line were seeded onto culture plates, and phytoestrogens apigenin (flavone), naringenin (flavanone), genistein (isoflavone), and quercetin (flavonol) were added after 24 h. 3-day incubation of cells with naringenin was found to have almost no antiproliferative effects. Genistein had a stronger proliferative effect and at the dose of 50 μM caused a 40% reduction in the number of living cells. Quercetin, a member of the flavonol group, exhibited a genistein-like activity. The highest antiproliferative effect was observed for apigenin (*Fig. 2A*) at a concentration of 50 μM (according to the MTT test data, 20% of MCF-7 cells compared with the control).

The ERα-negative SKBR3 cell line was used to answer the question of the possible impact of ERα expression on cell sensitivity to the antiproliferative action of phytoestrogens (at high concentrations). The distribution of SKBR3 cells by sensitivity to various phytoestrogens was similar to the distribution of ERα-positive MCF-7 cells. Naringenin was the least cytotoxic. Genistein and quercetin had a moderate antiproliferative effect. The highest antiproliferative activity was observed for apigenin: at a concentration of 50 μM, it caused the death of 60% of SKBR3 cells (3-day incubation with phytoestrogens, *Fig. 2B*). It should be noted that only quercetin (MCF-7 cells) and apigenin (MCF-7 and SKBR3 cells) reached the IC_50_ level (*Table*) after incubation of the cells with the phytoestrogens in the given range of concentrations (up to 50 μM). Therefore, naringenin and genistein are rather “weak” antiproliferative agents, and they should be tested in combination with compounds from other classes, e.g., antiestrogens of the SERM group (tamoxifen, etc.) and specific inhibitors of tyrosine kinases. Comparison of the number of viable MCF-7 and SKBR3 cells after 3-day incubation with 50 μM apigenin demonstrated that the SKBR3 line is more resistant to the cytostatic effect of apigenin than MCF-7 (40 and 20% of cells compared to the control, respectively,* p * < 0.05). On the basis of this observation, we presumed that high doses of apigenin could inhibit both the estrogen receptor signaling pathway (important factor for the growth of MCF-7 cells) and receptor tyrosine kinases, in particular HER2/neu (overexpression of this receptor was detected in SKBR3 cells).

**Table:** IC_50_ of phytoestrogens

IC_50_, μM	Naringenin	Genistein	Quercetin	Apigenin
MCF-7	> 50	> 50	50	25
SKBR3	> 50	> 50	> 50	30

The results of this series of experiments indicate that apigenin has the maximum antiproliferative effect among the tested phytoestrogens. Therefore, we further examined the molecular mechanisms of the action of high doses of this phytoestrogen on breast cancer cells.

**Effect of apigenin on the estrogen receptor activity**

**Fig. 3:**
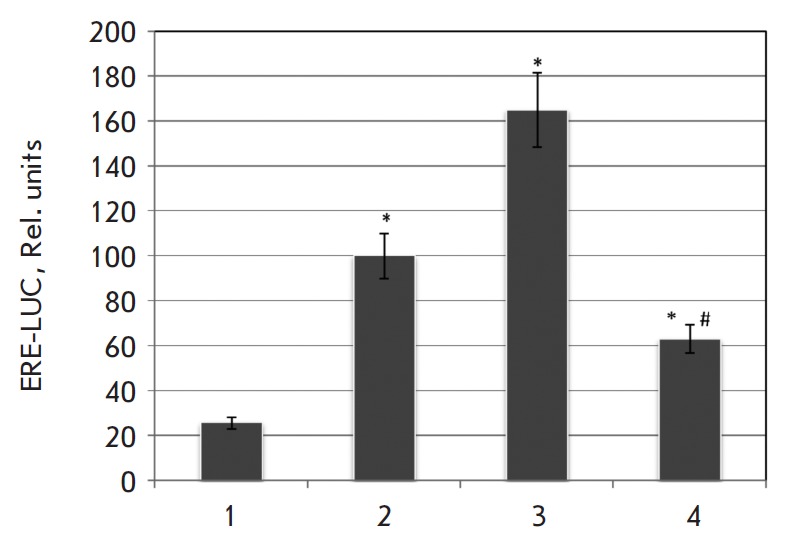
The effect of apigenin on the 17β-estradiolinduced activity of the estrogen receptor. After transfection with a reporter plasmid, MCF-7 cells were seeded onto 24-well plates and after 24 h were treated with 17β-estradiol and apigenin (1 – control MCF-7 cells; 2 – 10 nM 17β-estradiol; 3 – 10 nM 17β-estradiol and 5 μM apigenin; 4 – 10 nM 17β-estradiol and 50 μM apigenin). The luciferase activity was measured after 7 h of cultivation in the presence of the phytoestrogens according to the standard protocol by the reagent’s manufacturer (Promega, USA). ^*^p < 0.05 compared with control cells; #*p* < 0.05 for comparing columns 4 and 3

The tendencies discussed in the previous section indicate that the antiproliferative effect of phytoestrogens on breast cancer cells increases as their concentration increases. It is important to note that this effect is independent of the hormonal status of cells; however, the ERα-positive MCF-7 line is more sensitive to the antiproliferative action of high doses of apigenin (50 μM) than the ERα-negative SKBR3 line. We assumed that the increase in the concentration of apigenin is accompanied by a “switching-off” of the hormonal component of its action on breast cancer cells. To test this hypothesis, MCF-7 cells were transfected with a plasmid containing a reporter construct with the luciferase gene under the control of an estrogen-sensitive promoter. The cells were then transferred to a DMEM medium without phenol red (PanEco, Russia) and cultivated with addition of a 10% steroid-free fetal calf serum (HyClone, USA) for 24 h. The luciferase activity was measured after 7 h of cell growth in the presence of 17β-estradiol and apigenin. As shown in *Fig. 3*, lowdose apigenin had an estrogen-like effect and enhanced the inducing effect of 17β-estradiol on the estrogen receptor. A 10-fold increase in the apigenin concentration (to 50 μM) had the opposite effect: the phytoestrogen inhibited the estrogen receptor activity and prevented the action of 17β-estradiol. Thus, the anti-estrogenic properties of apigenin may be one of the explanations for the cytostatic effects of its high (50 μM) doses. These findings partly explain the effect of apigenin on MCF-7 cells: apigenin blocks the main proliferative stimulus for this tumor line. Which “target” does apigenin block in a ERα-negative SKBR3 breast cancer cell line? This issue was examined in the next series of experiments.

**Changes in the HER2/neu level during incubation of breast cancer cells with apigenin**

**Fig. 4:**
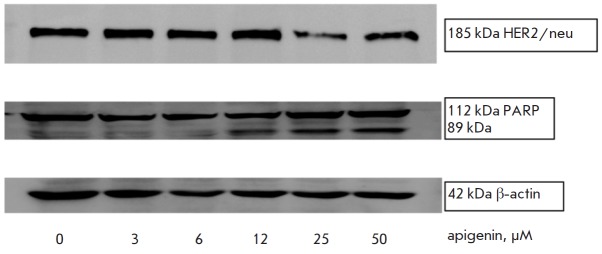
The effect of apigenin on HER2/neu expression and PARP degradation in SKBR3 cells. SKBR3 cells were treated with the apigenin concentrations shown in the figure for 3 days. The results of one of three independent experiments are shown

The expression of HER2/neu is known to be detected in 10–30% of breast cancers, which is regarded as a marker of poor prognosis [28, 29]. We analyzed the effect of apigenin on the HER2/neu expression in SKBR3 cells that produce this protein in sufficient quantities. As seen in *Fig. 4*, apigenin at concentrations from 3 to 12 μM does not affect the HER2/neu level in SKBR3 cells. However, incubation of the cells with higher doses of apigenin (25 and 50 μM) results in significant inhibition of the HER2/neu expression. Immunoblotting with antibodies to the apoptosis effector substrate, PARP, revealed partial degradation of PARP (identified as accumulation of a truncated 89 kDa form of PARP) upon increasing the apigenin concentration in SKBR3 cells.

The ability of phytoestrogens to lower the HER2/ neu level in tumor cells was discovered by Mai *et al*. [30] during incubation of the human breast cancer BT-474 cell line (HER2/neu+, ERα+) with 25 μM genistein. In addition, cultivation of BT-474 cells with genistein and an antiestrogen tamoxifen led to a further decrease in the expression of HER2/neu. A similar effect was observed for another member of the HER receptor family, EGFR (HER1) [30]. The phosphorylation level of HER2/neu and EGFR kinases was not analyzed, because the biological effect of genistein in this case was caused by a decrease in the level of its target protein (rather than by its activity). Sakla *et al*. [31] confirmed the data on the reduction in the HER2/neu level [30] and also showed that even at low doses (1 μM) genistein decreases the level of HER2/neu phosphorylation in BT-474 cells. Our data on a decrease in the HER2/ neu level in SKBR3 cells upon incubation with apigenin are consistent with the results obtained in another cell model (MDA-MB-453 breast cancer line) [32]. It was shown that the phytoestrogens apigenin, luteolin, naringenin, eriodictyol, and hesperetin at high doses (40 μM) cause degradation of HER2/neu in MDA-MB-453 cells. Initiation of apoptosis upon incubation of the cells with apigenin was found to occur through the release of cytochrome *c *and activation of caspase 3. Summarizing our findings and published data, we conclude that high doses of apigenin reduce the expression of one of the major tyrosine kinases supporting the growth of HER2-positive cells and simultaneously initiate apoptotic processes.

## CONCLUSIONS

The cytotoxic and antiproliferative effects of phytoestrogens on malignant cells are being extensively studied today [14, 33-38]. The interest in phytoestrogens is largely based on their natural origin and the relatively low cost of their synthesis and purification. In addition, there are data that support the prospects of their use for the prevention of cancer [38, 39]. Our work focuses on the investigation of the properties of flavone apigenin that exhibits a high antiproliferative activity in cells with various statuses of estrogen receptors. At high doses, apigenin was demonstrated to prevent the activation of the estrogen receptor by 17β-estradiol and cause inhibition of the HER2/neu expression, accompanied by a degradation of PARP in HER2-positive breast cancer cells. Other apigenin targets were identified in breast cancer cells, including proteins supporting the growth and survival of the tumor: PI3K/ Akt [40], STAT3 [33], NF-κB [34], p53 [34, 41], p21 [41], JAK3 [42], cyclins D1, D3, and Cdk4 [43]and VEGF [44]. Apparently, apigenin is a multi-target compound that triggers breast cancer cell death through the inhibition of receptor tyrosine kinases, decreased expression of growth factors, activation of p53, and suppression of key transcription factors. In 2008, a phase II clinical trial (NCT00609310) of a drug containing 20 mg of apigenin and 20 mg of epigallocatechin gallate in patients with colorectal cancer was registered on the Clinical- Trials.gov database. The first batch of data from this study, regarding changes in the disease relapse rate in patients treated with a mixture of these phytoestrogens, is expected in 2016. No other clinical trials of apigenin (as an antitumor agent) are currently registered on ClinicalTrials.gov. Further investigation of the antitumor activity of apigenin and its synthetic derivatives is quite promising, particularly in relation to HER2- positive breast tumors.
